# Olfactory Response of *Sitophilus zeamais* Adults to Odours of Semolina Pasta and Semolina Pasta Enriched with Different Amounts of *Acheta domesticus* Powder

**DOI:** 10.3390/insects15090634

**Published:** 2024-08-25

**Authors:** Pasquale Trematerra, Marco Colacci, Maria Cristina Messia, Maria Carmela Trivisonno, Anna Reale, Floriana Boscaino, Giacinto Salvatore Germinara

**Affiliations:** 1 Department of Agricultural, Environmental and Food Sciences, University of Molise, Via de Sanctis, I-86100 Campobasso, Italy; trema@unimol.it (P.T.); messia@unimol.it (M.C.M.); mariacarmela.trivisonno@unimol.it (M.C.T.); 2 Institute of Food Sciences, National Research Council, Via Roma 64, I-83100 Avellino, Italy; anna.reale@isa.cnr.it (A.R.); floriana.boscaino@isa.cnr.it (F.B.); 3 Department of Agricultural Sciences, Food, Natural Resources and Engineering, University of Foggia, Via Napoli 25, I-71122 Foggia, Italy; giacinto.germinara@unifg.it

**Keywords:** *Sitophilus zeamais*, semolina pasta, *Acheta domesticus* powder, olfactometric tests

## Abstract

**Simple Summary:**

The house cricket, *Acheta domesticus*, is one of the species recently authorised for production and commercialisation as novel food by European legislation. Powder from this insect can be used to enrich bread, extruded snacks, and pasta. The aim of this work was to analyse the behavioural response of one of the main pests of alimentary pasta, the maize weevil (*Sitophilus zeamais*), to different types of semolina pasta enriched or not enriched with increasing proportions of house cricket powder. In five-choice behavioural bioassays, the number of maize weevil adults attracted to 100% durum wheat pasta was significantly higher than those attracted to the other pasta types enriched with house cricket powder. In two-choice behavioural bioassays, all types of pasta had a positive and significant attraction. In similar experiments, cricket powder alone was not attractive, indicating a neutral effect towards insects. These observations suggest that the lower attractiveness of pasta enriched with cricket powder is mainly due to a masking effect of host food odours.

**Abstract:**

The behavioural response of adult maize weevil, *Sitophilus zeamais*, to different types of semolina pasta enriched or not enriched with increasing proportions (5%, 10%, and 15%) of house cricket (*Acheta domesticus*) powder was investigated in olfactometer arena bioassays by using trap devices. In the five-choice behavioural bioassays, the number of *S. zeamais* adults attracted to 100% durum wheat semolina pasta was significantly higher than those attracted to the other pasta types enriched with *A. domesticus* powder. In the two-choice behavioural bioassays, the Response Index for each pasta type was positive and significant. However, although not significant, there was a progressive reduction in the Response Index as the cricket powder content increased. In similar experiments, there were no significant differences between cricket powder alone and the control in the number of attracted *S. zeamais*, indicating a neutral effect towards insects. These observations suggest that the lower attractiveness of pasta enriched with house cricket powder is mainly due to the masking of host food odours. Solid-phase microextraction coupled to gas chromatography–mass spectroscopy identified a total of 18 compounds in the head-space samples of the different types of pasta, highlighting differences in volatile composition. Some volatile compounds were only present in the pasta produced with cricket powder. In particular, 1-octen-3-ol and phenol were present in the samples containing 5%, 10%, or 15% cricket powder; pentanal, benzaldehyde, and dimethyl disulphide were present in samples containing 10% or 15% cricket powder; and 2,5-dimethyl-pyrazine was present in the sample containing 15% cricket powder. Further investigation with individual compounds and mixtures is needed to define the chemical basis of the differences in the insect olfactory preference observed in this study.

## 1. Introduction

Policy makers, researchers, and the food industry have focused their attention on the use of insects in the formulation of foods because of their nutritional value (high protein content, essential amino acids, polyunsaturated fatty acids, minerals, and vitamins) [1,2,3,4,5,6].

In this context, the market and consumers also demand new types of pasta with healthy nutritional characteristics (e.g., a good source of protein and fibre, gluten-free pasta, and pasta made with non-genetically modified organisms). In an attempt to achieve this goal, researchers have evaluated the enrichment of cereal products with insect powder [5,7,8].

The consumption of insects in Western countries is currently very limited due to consumer neophobia and low acceptability, but their inclusion within other foods (e.g., in dried pasta), instead of the consumption of insects in their normal form, could help to increase their acceptability.

*Acheta domesticus* (L.) (Orthoptera: Gryllidae) is one of the species recently authorised by European legislation (Commission Implementing Regulation (EU) 2023/5 of 3 January 2023 amends Implementing Regulation (EU) 2017/2470) for production and commercialisation. This Regulation allows for the placing of *A. domesticus* (house cricket) partially defatted powder on the market as a novel food [9].

Pasta can be infested, during the entire production and distribution chain, by several insects with negative economic and commercial consequences [10,11,12,13].

Among other insects, weevils (*Sitophilus* spp.) are universally regarded as one of the most destructive primary pests of stored cereals [14]; they can also infest split peas and various types of pasta [15,16]. In particular, the maize weevil, *Sitophilus zeamais*, follows the odour of pasta based on volatile organic compounds (VOCs) and can enter packages of commercial products (e.g., rice or pasta) [10,13,15,16,17,18].

Most commercially available alimentary products are packaged to prevent infestation, although insect contamination is still frequent. This results in serious economic damage to food companies. To prevent infestation, resistant and sealed packages have been proposed [19,20,21,22,23,24,25,26].

In the present work, we investigated the relationships between *Sitophilus zeamais* and pasta without and with *A. domesticus* powder. To our knowledge, there is no published study that has considered *S. zeamais* infestations in durum wheat semolina dry pasta enriched with different levels of *A. domesticus* powder [27]. Because olfactory stimuli play an important role in *S. zeamais* infestation of food sources, we investigated, in an olfactometer arena, the behavioural responses of adult maize weevils to the odours of four types of cereal pasta, three of which were enriched with different amounts of *A. domesticus* powder, to understand their biological activity. In addition, we analysed headspace solid-phase microextraction (HS-SPME) extracts from the four types of pasta tested and for the *A. domesticus* powder by gas chromatography coupled to mass spectrometry (GC-MS) to determine amount between their odour compounds.

## 2. Materials and Methods

### 2.1. Insects

A wild *S. zeamais* population, found in June 2023 on barley on a livestock farm (Bojano, Molise, Italy), was used for laboratory rearing (climatic chamber at 28 ± 2 °C, 70% ± 5% relative humidity, and a 12:12 L:D photoperiod). Unsexed 1- to 2-week-old adults (F1 population) were used in the behavioural bioassays.

### 2.2. Pasta

#### 2.2.1. Pasta Raw Materials

Durum wheat (*Triticum durum* Desf.) semolina (Casillo Group, Corato, Bari, Italy) and partially defatted commercial powder of *A. domesticus* cricket (S.A. Italian Cricket Farm, Scalenghe, Torino, Italy) were used to produce pasta. The nutritional values of the durum wheat semolina and the partially defatted cricket powder, as reported by the resellers, are shown in Table 1.

#### 2.2.2. Pasta Making

The mixing of the flours and dough extrusion were carried out with a “La Perfetta medium” pasta machine (La Prestigiosa, Villaverla, Vicenza, Italy). The extruded pasta was dried below 80 °C by using temperature–moisture equilibrium curves. All pasta samples were dried at the same time.

Specifically, four different types of pasta (samples A–D) were produced and used for the behavioural bioassays: 100% durum wheat semolina (A); 95% durum wheat semolina + 5% cricket powder (B); 90% durum wheat semolina + 10% cricket powder (C); and 85% durum wheat semolina + 15% cricket powder (D). The chemical composition of the pasta types is shown in Table 2.

The moisture, ash, fat and protein contents of the pasta were determined according to ICC methods 109/1, 104/1, 117, and 105/2, respectively [28]. Dietary fibre was determined according to AACC method 32.05 [29].

### 2.3. Five-Choice Behavioural Bioassay

Five-choice behavioural bioassays were carried out in cylindrical Plexiglas arenas (80 cm diameter × 40 cm height). In each arena, five modified Flit-Track M2 trap devices (Trécé Incorporated, Adair, OK, USA) [13] were placed; four trap devices contained 6 g of the four types of pasta [100% durum wheat semolina (A); 95% durum wheat semolina + 5% cricket powder (B); 90% durum wheat semolina + 10% cricket powder (C); 85% durum wheat semolina + 15% cricket powder (D)], and one trap device was left empty, used as a control (E). Teflon paint was applied to the internal vertical surface of the arena and of the trap devices to prevent the maize weevils from escaping.

In each trial, 100 *S. zeamais* adults of mixed sexes were released at the centre of the arena [15]. One day and seven days after their introduction into the arena, the number of trapped insects was checked. For each experiment (at 1 day and at 7 days), twelve replicates were performed, using a total of 2400 insects. In all replications, the trap device positions were randomised and their contents renewed. The tests were carried under the same conditions of the rearing climatic chamber (see Section 2.1).

### 2.4. Two-Choice Behavioural Bioassay

To evaluate the attractiveness of adult maize weevils to each type of pasta, two-choice behavioural bioassays were carried out in cylindrical olfactometer arenas, using the modified Flit-Track M2 trap devices and the methodology described above for the five-choice behavioural bioassays. Similarly to the pasta samples, 6 g of *A. domesticus* powder was used. The following pairwise comparisons were performed: 100% durum wheat semolina vs. control (samples A vs. E); 95% durum wheat semolina + 5% cricket powder vs. control (samples B vs. E); 90% durum wheat semolina + 10% cricket powder vs. control (samples C vs. E); 85% durum wheat semolina + 15% cricket powder vs. control (samples D vs. E); cricket powder vs. control (samples F vs. E).

The number of trapped insects in baited and unbaited (control) trap devices was checked 1 day and 7 days after their introduction into the arena. For each experiment, there were 5 replicates, using a total of 5000 insects. In each trial, the Response Index (RI) was evaluated by using the following formula: RI = [(T − C)/Total] × 100, where T is the number of samples responding to the bait trap, C is the number of insects responding to the control (unbaited trap), and Total is the total number of insects released in the arena [17].

### 2.5. HS-SPME-GC/MS Analysis of Volatile Components

The *A. domesticus* powder and each type of pasta were subjected to VOC analysis. The volatile fraction of the samples was extracted by using HS-SPME, followed by analysis with GC-MS, according to Zotta et al. [30].

SPME-GC/MS was performed by using an Agilent GC 7890A/MSD 5975 system with a Gerstel MPS2 automatic sampler (Agilent Technologies, Santa Clara, CA, USA).

VOCs were identified by comparing mass spectra with the National Institute of Standards and Technology (NIST) library (NIST 20) with match factor ≥80%. Whenever possible, mass retention times and mass spectra of tentatively identified compounds by using the NIST library were compared with those of commercially available authentic standards. The retention index was calculated according to the equation reported by Van Den Dool and Kratz (1963) [31]. Then, it was compared with a series of alkanes by using the online NIST database (http://webbook.nist.gov/chemistry/; accessed on 1 September 2023) for high polar column for InnoWAX or similar stationary phases. All the compounds were identified by the matching of the retention indices and mass spectra. The results are expressed as the relative peak area (RAP), calculated as follows: area peak compound/area peak internal standard × 100. Each value is expressed as the mean ± standard deviation.

Blank experiments were conducted in two different modalities: blank fibre and a blank empty vial. The analyses were performed in duplicate.

### 2.6. Data Analysis

Friedman two-way analysis of variance (ANOVA) by ranks was used to analyse the number of *S. zeamais* adults found in the different trap devices in the five-choice behavioural bioassays. When *p* < 0.05, means were separated by using the Wilcoxon signed ranks test.

Student’s *t*-test for paired comparisons was used to compare the mean number of insects found for the two-choice behavioural bioassays. One-way ANOVA followed by Tukey’s HSD post hoc test was used to compare the mean RIs.

SPSS Statistics 13.0 (SPSS Inc., Chicago, IL, USA) was used to perform the statistical analyses.

Principal Component Analysis (PCA) was performed on the VOC profiles by using Tanagra 1.4 software.

## 3. Results

### 3.1. Five-Choice Behavioural Bioassay

**Figure 1:**
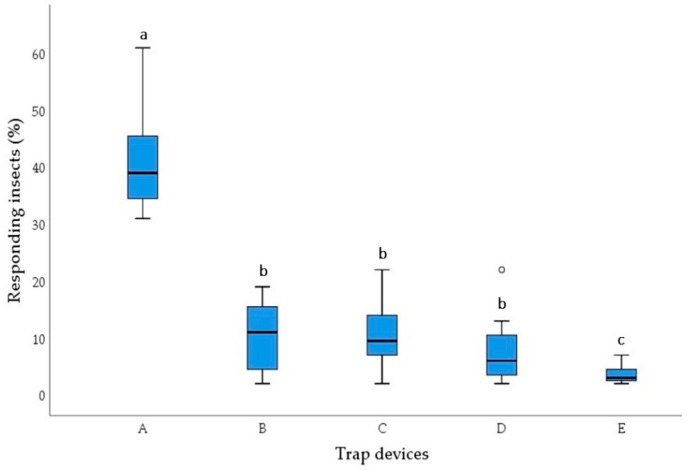
Behavioural responses of *Sitophilus zeamais* adults to 100% durum wheat semolina pasta (A), pasta with 95% durum wheat semolina + 5% cricket powder (B), pasta with 90% durum wheat semolina+ 10% cricket powder (C), pasta with 85% durum wheat semolina + 15% cricket powder (D), and control (E) in 1-day five-choice bioassays. Each box plot indicates the median and its range of dispersion (lower and upper quartiles and outliers). Above each box plot, different letters indicate significant differences (Wilcoxon test, *p* < 0.05).

**Figure 2:**
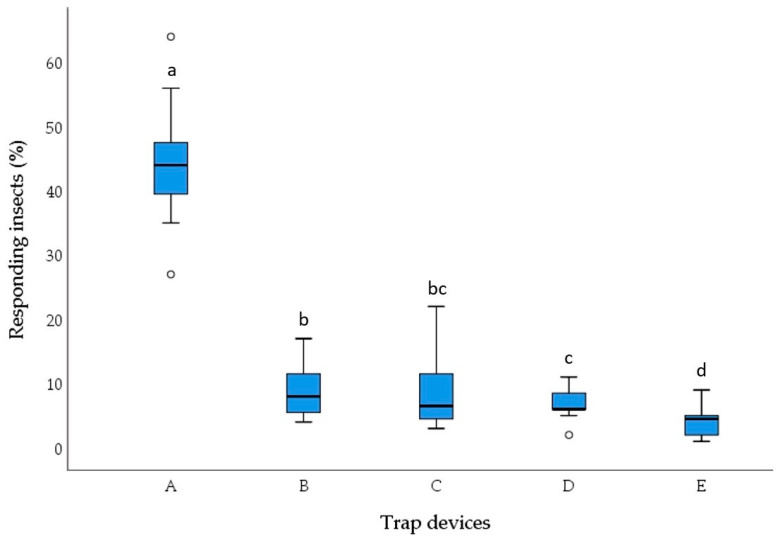
Behavioural responses of *Sitophilus zeamais* adults to 100% durum wheat semolina pasta (A), pasta with 95% durum wheat semolina + 5% cricket powder (B), pasta with 90% durum wheat semolina+ 10% cricket powder (C), pasta with 85% durum wheat semolina + 15% cricket powder (D), and control (E) in 7-day five-choice bioassays. Each box plot indicates the median and its range of dispersion (lower and upper quartiles and outliers). Above each box plot, different letters indicate significant differences (Wilcoxon test, *p* < 0.05).

The behavioural responses of *S. zeamais* adults to the four types of pasta in multi-choice olfactometer bioassays are reported in Figure 1 and Figure 2. There were significant differences in the number of adults found in the trap devices containing different pasta types 1 day (χ^2^ = 33.017, df = 4, *p* < 0.001) and 7 days after the start of the experiment (χ^2^ = 31.162, df = 4, *p* < 0.001).

The 1-day and 7-day experiments revealed similar results. *S. zeamais* showed a significant preference for pasta made with 100% durum wheat semolina compared with pasta enriched with cricket powder. In fact, trap devices baited with 100% durum wheat semolina pasta (A) captured an average of 40.75% and 44.25% of the adults released into the olfactometer arena in the 1-day and 7-day experiments, respectively. The number of insects attracted to 100% durum wheat semolina pasta (A) was significantly higher than the number attracted to the three types of pasta (B, C, and D) enriched with cricket powder (Wilcoxon test, *p* < 0.05). The number of insects captured by the control trap device (E) was significantly lower compared with those attracted to the four pasta samples (Wilcoxon test, *p* < 0.05).

Statistical differences (Wilcoxon test, *p* < 0.05) among the three pasta types enriched with cricket powder were found in the 7-day experiment but not in the 1-day experiment.

After 7 days, among the three pasta types enriched with cricket powder, the number of insects attracted to sample D, containing the highest amount of cricket powder, was significantly lower than the number of insects attracted to sample B, containing the lowest amount of cricket powder (Wilcoxon test, *p* < 0.05). Sample C, made with an intermediate amount of cricket powder, showed no significant differences compared with samples B and D (Wilcoxon test, *p* < 0.05).

### 3.2. Two-Choice Behavioural Bioassay

The results of the two-choice behavioural bioassays are reported in Table 3 and Table 4. When tested individually, 100% durum wheat semolina (A), 95% durum wheat semolina + 5% cricket powder (B), 90% durum wheat semolina + 10% cricket powder (C), and 85% durum wheat semolina + 15% cricket powder (D) pasta types attracted a significantly higher number of adult maize weevils compared with an empty, modified Flit-Track M2 trap device used as a control (A vs. E; B vs. E; C vs. E; D vs. E) in both the 1-day and 7-day experiments. There was a significant difference when comparing the RI for each type of different pasta types and cricket powder in the 1-day (ANOVA: F = 35.223, df = 4, *p* < 0.001) and in 7-day (ANOVA: F = 19.263, df = 4, *p* < 0.001) experiments.

The mean RI did not differ significantly among the types of pasta, and all were positive and significant, indicating actual attraction of maize weevils. However, the RI decreased from 56.8 ± 3.4 and 60.0 ± 4.5 in response to 100% semolina pasta in the 1-day and 7-day experiments, respectively, to 48.4 ± 1.9 and 42.8 ± 5.2 for pasta containing the highest amount of cricket powder, suggesting an inverse relationship between the attractiveness of the pasta sample and the cricket powder content. On the contrary, there was a non-significant RI for the trap devices baited with cricket powder compared with the empty trap device (F vs. E) in the 1-day and in 7-day experiments, indicating a neutral effect on maize weevil orientation.

### 3.3. HS-SPME-GC/MS Analysis of Volatile Components

Table S1 shows the volatile composition of pasta made of 100% durum wheat semolina (sample A), and pasta made with different concentrations of partially defatted powder of cricket *A. domesticus* (samples B–D) and of the cricket powder used to produce pasta (sample F).

The analysis identified 18 compounds in the pasta samples: three ketones, four aldehydes, one ester, two alcohols, one acid, one sulphur compound, one pyrazine, one terpene, two furan compounds, and two other compounds.

Some volatile compounds were only present in the pasta produced with cricket powder. In particular, 1-octen-3-ol and phenol were present in all three samples containing cricket powder (B, C, and D); pentanal, benzaldehyde, and dimethyl disulphide were present in the samples containing 10% or 15% cricket powder (C and D); and 2,5-dimethyl-pyrazine was in the sample containing 15% cricket powder (D).

In contrast, pasta made with 100% wheat flour (A) had higher amounts of 2,6-dimethyl-4-heptanone, 2-octanone, 2-hexenal and β-myrcene than the samples produced with cricket powder.

Furthermore, pasta made with 100% semolina (sample A) and pasta containing cricket powder (B, C, and D) differed mainly in 1-octen-3-ol, ethyl acetate, dimethyl disulphide, and phenol.

The SPME/GS-MS analysis of the cricket powder (sample F) identified more than 40 volatile compounds. The compounds belonged to eight main classes, namely, ketones (seven), aldehydes (four), esters (one), alcohols (three), acids (six), sulphur compounds (four), pyrazines (five), and furan compounds (two). There were also three compounds from other classes.

The most abundant compounds were alcohols, ketones, and acids, while moderate amounts of aldehydes, pyrazines, and furan compounds were detected. Traces of esters and sulphur compounds were found. Alcohols and acids were mainly represented by ethanol and acetic acid, respectively. Among the ketones, 2-heptanone and 2-propanone were the most abundant. Hexanal, 3-methyl-butanal, benzaldehyde and pentanal were the aldehydes found. Dimethyl disulphide, 2,5-dimethyl-pyrazine, and 2-pentyl-furan were the most abundant sulphur, pyrazine, and furan compounds, respectively. Ethyl acetate was the only ester found. Other compounds found were toluene and acetamide.

**Figure 3:**
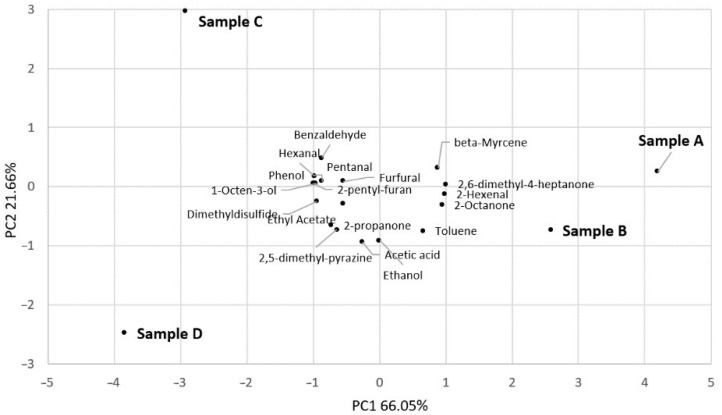
Principal Component Analysis (PCA) of volatile organic compounds that mainly differentiated the four samples of pasta: 100% durum wheat semolina (A); 95% durum wheat semolina + 5% cricket powder (B); 90% durum wheat semolina + 10% cricket powder (C); 85% durum wheat semolina + 15% cricket powder (D).

We performed a PCA to better understand the differences between the pasta made with 100% semolina and pasta made with different amounts of *A. domesticus* powder (Figure 3). The first two principal components (PCs) explained 87.71% of the total variance in the data. Based on the first two PCs (factors), the samples had a different volatile composition. In particular, sample A, produced with 100% durum wheat semolina, was located in the I square, while the samples produced with the addition of 5% (sample B), 10% (sample C), and 15% (sample D) cricket powder where located in the IV, II, and III squares, respectively. The main separation was found between sample A produced with 100% durum wheat semolina (positively associated to PC1) and samples C and D produced with 10 and 15% cricket powder (negatively associated to PC1), respectively. In particular, the samples with the highest contents of cricket powder (10% and 15%) differed from sample A in benzaldehyde, dimethyldisulphide, ethyl acetate, phenol, and 2-pentyl-furan.

## 4. Discussion

As mentioned earlier, the most common and dangerous species that can infest pasta are those of the genus *Sitophilus*. The susceptibility to infestation of the various types of pasta found on the market has been studied on several occasions, with results that are of practical operational interest to food companies [13,15,16].

In the present study, we evaluated four types of pasta made of 100% durum wheat semolina or containing increasing proportions of cricket powder (5%, 10%, and 15%) regarding their ability to attract maize weevil adults in an olfactometer arena. Multi-choice and two-choice behavioural bioassays, lasting 1 day and 7 days, were performed. For both durations of the experiments, similar results were found, as also reported in Trematerra et al. 2021 [15].

In the multi-choice behavioural bioassays, trap devices baited with pasta made with 100% durum wheat semolina, captured a significantly higher number of adult maize weevils compared with the other three pasta types enriched with *A. domesticus* powder. These data demonstrate that under multi-choice conditions, maize weevil adults avoided pasta types enriched with cricket powder when offered in the presence of 100% durum wheat semolina pasta.

In the two-choice tests, maize weevils had to choose between a trap baited with one pasta sample and an empty trap used as a control. Under that more restrictive condition of choice, maize weevils exhibited a significant olfactory preference for all types of pasta, including those enriched with cricket powder. However, even if not significantly different, the RI decreased as the cricket powder content increased. Overall, the lower attractiveness of pasta enriched with cricket powder appears to be due to an alteration in the attractive blend emitted by wheat semolina pasta by cricket powder volatile compounds. This hypothesis is also supported by the neutral effect of cricket powder on the olfactory orientation of *S. zeamais* adults, as evidenced by the non-significant RI recorded for cricket powder alone in the two-choice behavioural bioassays.

The antennae of *S. zeamais* are capable of selectively perceiving a wide range of cereal volatile compounds [32]. The importance of the ratios and concentrations of volatile compounds in host location by phytophagous insects has been demonstrated [33,34,35].

It is worth noting that some VOCs identified in the head-space samples of cricket powder in this study have been shown to be bioactive towards stored-product insect pests, including *S. zeamais*. These compounds include dimethyl disulphide and benzaldehyde. Dimethyl disulphide exerts insecticidal neurotoxicity through mitochondrial dysfunction and activation of insect KATP channels [36]. Park et al. (2005) [37] and Huang et al. (2000) [38] reported fumigant toxicity of this compound against the Japanese termite, *Reticulitermes speratus* (Kolbe), and stored product pests, *S. zeamais* and *Tribolium castaneum* (Herbst). Similarly, benzaldehyde was reported to be a safer fumigant to control *Sitophilus oryzae* (L.) and *T. castaneum* [39,40] Therefore, it also seems likely that the perception of these substances, known to be toxic at high doses, may have induced maize weevils to move away from a source of toxic compound [41], resulting in avoidance of pasta types containing cricket powder.

According to Nordlund and Lewis (1976) [42], the volatile message that *S. zeamais* adults receive seems to show interspecific action such as apneumone-like biological allelochemical activity. *S. zeamais* adults seem to recognise in the volatile message the presence of substances that stimulate interspecific cannibalism/necrophagy, a behaviour this species does not possess, as it has evolved and specialised over time in plants, particularly cereals [43,44,45]. Further investigation is needed to assess whether these interactions are responsible for the differences in the behavioural responses.

The present study revealed that partially defatted powder of cricket *A. domesticus* can be successfully included in pasta products to increase their protein content. This research also responds to the growing consumer awareness of healthy products by demonstrating that edible insects can be a new source of innovative ingredients for use in pasta production. Given the aminoacidic composition of insect protein, it could be a promising ingredient to improve the biological value of pasta protein, which is generally low in lysine.

## 5. Conclusions

The results of this study revealed that cricket powder and semolina pasta enriched with cricket powder have a neutral effect on *S. zeamais* adult choice behaviour. The lower choice for pasta enriched with cricket powder compared with pasta produced with 100% semolina could be due to a masking effect of host food odours. Further investigation is needed to assess the reason for the choice of the different types of pasta used in the trials.

## Figures and Tables

**Table 1:** Nutrition facts (g/100 g) of durum wheat semolina and commercially available partially defatted *Acheta domesticus* powder.

Values	Durum Wheat Semolina	Cricket Powder
Energy value	1446 kJ/346 kcal	1738 kJ/415.4 kcal
Fat	2.0 g	9.0 g
Carbohydrates	67.0 g	5.0 g
Fibre	2.8 g	9.2 g
Protein	13.5 g	74.0 g
Salt	0.005 g	1.1 g

**Table 2:** The chemical composition (g/100g) of the dry pasta produced with durum wheat semolina and different percentages of partially defatted powder of house cricket *Acheta domesticus*. [100% durum wheat semolina (A); 95% durum wheat semolina + 5% cricket powder (B); 90% durum wheat semolina + 10% cricket powder (C); 85% durum wheat semolina + 15% cricket powder (D)].

Samples	Moisture	Fat	Carbohydrates *	Dietary Fibre	Protein (N*6.25)	Ash
A	10.6	2.1	69.5	2.9	14.2	0.73
B	10.6	2.4	69.9	3.2	17.0	0.89
C	10.6	2.8	62.2	3.5	19.9	1.04
D	10.6	3.1	58.6	3.8	22.7	1.19

* Calculated by difference.

**Table 3:** Behavioural response of *Sitophilus zeamais* adults reported as mean absolute number (±Standard Error) caught in bait trap devices and in unbaited control and mean Response Indices in the 1-day two-choice bioassays. In each row, significant differences between baited trap devices and the unbaited control were determined by Student’s *t*-test. [100% durum wheat semolina (A); 95% durum wheat semolina + 5% cricket powder (B); 90% durum wheat semolina + 10% cricket powder (C); 85% durum wheat semolina + 15% cricket powder (D); unbaited control (E); and 100% cricket powder (F)].

Samples	Bait Trap Devices (±SE)	Unbaited Control (±SE)	Student’s *t*-Test		Response Index (±SE)
			t-Value	*p*-Value	
A vs. E	62.4 ± 3.0	5.6 ± 1.6	16.591	<0.001	56.8 ± 3.4 a
B vs. E	54.6 ± 4.5	4.8 ± 0.9	10.919	<0.001	49.8 ± 4.0 a
C vs. E	56.0 ± 6.0	5.2 ± 1.7	8.172	<0.001	50.8 ± 5.9 a
D vs. E	53.2 ± 4.8	4.8 ± 2.2	12.033	<0.001	48.4 ± 1.9 a
F vs. E	10.0 ± 1.8	8.0 ± 1.5	0.853	0.419	2.0 ± 2.3 b

In the Response Index column, means followed by the same letter are not significantly different (Tukey’s HSD post hoc test, *p* < 0.001).

**Table 4:** Behavioural response of *Sitophilus zeamais* adults reported as mean absolute number (±Standard Error) caught in bait trap devices and in unbaited control and mean Response Indices in the 7-day two-choice bioassays. In each row, significant differences between baited trap devices and the unbaited control were determined by Student’s *t*-test. [100% durum wheat semolina (A); 95% durum wheat semolina + 5% cricket powder (B); 90% durum wheat semolina + 10% cricket powder (C); 85% durum wheat semolina + 15% cricket powder (D); unbaited control (E); and 100% cricket powder (F)].

Samples	Bait Trap Devices (±SE)	Unbaited Control (±SE)	Student’s *t*-Test		Response Index (±SE)
			t-Value	*p*-Value	
A vs. E	63.0 ± 4.1	3.0 ± 0.7	14.384	<0.001	60.0 ± 4.5 a
B vs. E	55.2 ± 4.8	5.4 ± 1.4	9.920	<0.001	49.8 ± 4.9 a
C vs. E	53.8 ± 7.2	3.6 ± 0.8	6.922	<0.001	50.2 ± 7.3 a
D vs. E	47.8 ± 4.9	5.0 ± 1.2	8.420	<0.001	42.8 ± 5.2 a
F vs. E	9.2 ± 2.1	7.6 ± 1.4	0.637	0.542	1.6 ± 3.4 b

In the Response Index column, means followed by the same letter are not significantly different (Tukey’s HSD post hoc test, *p* < 0.001).

## Data Availability

Data are contained within article.

## Supplementary Materials

The following supporting information can be downloaded at: https://www.mdpi.com/article/10.3390/insects15090634/s1, Table S1: Volatile compounds derived from pasta made of 100% durum wheat semolina (sample A), pasta made with different concentrations of partially defatted powder of cricket A. domesticus [pasta made with 95% durum wheat semolina + 5% cricket powder (sample B); pasta made with 90% durum wheat semolina + 10% cricket powder (sample C); pasta made with 85% durum wheat semolina + 15% cricket powder (sample D)] and cricket powder used to produce pasta (sample F).
