# Supplementary material for: Olfactory Response of Sitophilus zeamais Adults to Odours of Semolina Pasta and Semolina Pasta Enriched with Different Amounts of Acheta domesticus Powder
Source: Insects. 2024 Aug 25;15(9):634. doi: 10.3390/insects15090634 (PMC11431939; doi:10.3390/insects15090634)
Supplement: Supplementary file 1 [file insects-15-00634-s001.zip › insects-3135616-supplementary.pdf]

Table S1. Volatile compounds derived from pasta made of 100% durum wheat semolina (sample A), pasta made with different concentrations of partially defatted powder of cricket *A. domesticus* [pasta made with 95% durum wheat semolina + 5% cricket powder (sample B); pasta made with 90% durum wheat semolina + 10% cricket powder (sample C); pasta made with 85% durum wheat semolina + 15% cricket powder (sample D)] and cricket powder used to produce pasta (sample F).

Retention Index	Compounds	Mean value ± SD				
		Sample A	Sample B	Sample C	Sample D	Sample F
	Ketones					
800	2-propanone	0.54 ± 0.02	0.55 ± 0.06	0.60 ± 0.06	1.06 ± 0.19	27.12 ± 0.97
1048	2,3-pentanedione	0	0	0	0	0.93 ± 0.07
1140	2,6-dimethyl-4-heptanone	2.35 ± 0.17	2.03 ± 0.04	1.04 ± 0.12	0.79 ± 0.10	0
905	2-butanone	0	0	0	0	18.32 ± 1.74
1184	2-heptanone	0	0	0	0	69.10 ± 0.93
1270	2-octanone	2.36 ± 0.20	2.39 ± 0.10	0.36 ± 0.02	0.85 ± 0.07	5.69 ± 0.45
1378	2-nonanone	0	0	0	0	8.54 ± 0.79
1611	butyrolactone	0	0	0	0	2.97 ± 0.22
	Aldehydes					
915	3-methyl-butanal	0	0	0	0	11.34 ± 0.66
980	pentanal	0	0	0.16 ± 0.02	0.15 ± 0.01	7.49 ± 0.65
1061	hexanal	2.35 ± 0.35	2.39 ± 0.10	2.82 ± 0.37	2.78 ± 0.27	12.71 ± 0.92
1205	2-hexenal	3.55 ± 0.14	3.71 ± 0.12	0	0	0
1506	benzaldehyde	0	0	0.49 ± 0.02	0.27 ± 0.03	8.06 ± 0.41
	Ester					
893	ethyl acetate	0.44 ± 0.03	1.09 ± 0.05	0.90 ± 0.04	1.03 ± 0.07	2.82 ± 0.24
	Alcohols					
932	ethanol	688.16 ± 25.93	643.12 ± 34.09	566.47 ± 51.85	750.33 ± 84.06	1615.37 ± 31.98
1232	1-pentanol	0	0	0	0	9.13 ± 0.35
1420	1-octen-3-ol	0	0.27 ± 0.01	0.54 ± 0.04	0.53 ± 0.04	9.26 ± 0.30
	Acid					
1425	acetic acid	0.61 ± 0.05	0.64 ± 0.05	0.60 ± 0.01	0.66 ± 0.05	107.41 ± 0.36
1498	propanoic acid	0	0	0	0	6.50 ± 0.09
1531	isobutanoic acid	0	0	0	0	5.54 ± 0.22
1620	butanoic acid	0	0	0	0	1.86 ± 0.12
1640	isovaleric acid	0	0	0	0	3.58 ± 0.36
1720	pentanoic acid	0	0	0	0	0.30 ± 0.03
	Sulfur compound					
690	methanethiol	0	0	0	0	0.87 ± 0.08
1050	dimethyldisulfide	0	0	0.15 ± 0.01	0.27 ± 0.04	4.74 ± 0.23
1559	dimethyl sulfoxide	0	0	0	0	0.68 ± 0.08
1890	dimethyl sulfone	0	0	0	0	0.98 ± 0.03
	Pyrazine					
1252	methylpyrazine	0	0	0	0	10.30 ± 0.27
1318	2,5-dimethyl-pyrazine	0	0	0	0.11 ± 0.01	20.84 ± 1.38
1325	2,3-dimethyl-pyrazine	0	0	0	0	2.26 ± 0.14
1383	2-ethyl-6-methyl-pyrazine	0	0	0	0	4.70 ± 0.17
1401	trimethyl-pyrazine	0	0	0	0	7.54 ± 0.16
1467	3,5-diethyl-2-methyl-pyrazine	0	0	0	0	1.13 ± 0.07
	Terpene					
1130	β-myrcene	1.15 ± 0.11	0.87 ± 0.14	0.81 ± 0.06	0.64 ± 0.08	0
	Furan compounds					
1223	2-pentyl-furan	0.35 ± 0.03	0.40 ± 0.01	0.55 ± 0.05	0.56 ± 0.05	21.60 ± 2.20

1443	furfural	0.30 ± 0.02	0.13 ± 0.02	0.31 ± 0.01	0.34 ± 0.04	0
1635	2-furanmethanol	0	0	0	0	3.33 ± 0.14
	Others					
1030	toluene	0.57 ± 0.02	0.60 ± 0.09	0.31 ± 0.03	0.52 ± 0.04	21.02 ± 0.66
1774	acetamide	0	0	0	0	18.98 ± 0.60
1990	phenol	0	0.06 ± 0.01	0.09 ± 0.01	0.08 ± 0.01	0.32 ± 0.02

Retention indices were calculated with van Den Dool and Kratz formula. Calculated Retention indices were compared using the online NIST database (http://webbook.nist.gov/chemistry/; accessed on 1 September 2023) for high polar column for InnoWAX or similar stationary phases. All the compounds were identified by the matching Retention indices and MS. The results are expressed as RAP = relative peak area (area peak compound/area peak internal standard) * 100. Each value is expressed as mean ± SD.
